# Preoperative Radiochemotherapy in Esophageal Squamous Cell Cancer with 5-Fluorouracil/Cisplatin or Carboplatin/Paclitaxel: Treatment Practice over a 20-Year Period and Implications for the Individual Treatment Modalities

**DOI:** 10.3390/cancers13081834

**Published:** 2021-04-12

**Authors:** Leif Hendrik Dröge, Philipp Johannes Karras, Manuel Guhlich, Markus Anton Schirmer, Michael Ghadimi, Stefan Rieken, Lena-Christin Conradi, Martin Leu

**Affiliations:** 1 Department of Radiotherapy and Radiation Oncology, University Medical Center Göttingen, Robert-Koch-Str. 40, 37075 Göttingen, Germany; manuel.guhlich@med.uni-goettingen.de (M.G.); mschirmer@med.uni-goettingen.de (M.A.S.); Stefan.rieken@med.uni-goettingen.de (S.R.); martin.leu@med.uni-goettingen.de (M.L.); 2 Department of General, Visceral and Pediatric Surgery, University Medical Center Göttingen, 37075 Göttingen, Germany; p.karras@alexianer.de (P.J.K.); mghadim@uni-goettingen.de (M.G.); lena.conradi@med.uni-goettingen.de (L.-C.C.); 3 Department of General and Visceral Surgery, Raphaelsklinik, 48143 Münster, Germany

**Keywords:** esophageal cancer, preoperative radiochemotherapy, chemotherapy regimen, acute toxicity, hematologic toxicity, complications, prognostic factors, pathological complete response

## Abstract

**Simple Summary:**

We retrospectively studied outcomes in patients treated with preoperative radiochemotherapy and surgery for esophageal squamous cell cancer. We put a special focus on the comparison of patients treated with 5-fluorouracil/cisplatin (‘Walsh’) or carboplatin/paclitaxel (‘CROSS’). First, the higher age and more comorbidities of ‘CROSS’ patients, along with a shorter intensive care/intermediate care unit stay, might reflect an improvement in supportive and surgical/perioperative procedures in the periods. Second, the ‘CROSS’ patients experienced more hematologic toxicity and were less likely to complete chemotherapy as per protocol. This indicates that efforts should be taken to guide patients through a toxic treatment regimen. Third, the negative prognostic impact of radiochemotherapy-related toxicities and the duration of the intensive care/intermediate care unit stay underlines that further optimization of treatment procedures remains an important goal. Toxicity profiles could be improved by tailoring the regimen to individual patients (e. g., careful use of the taxane-based regimen in elderly patients).

**Abstract:**

We retrospectively studied outcomes in patients treated with preoperative radiochemotherapy and surgery for esophageal squamous cell cancer. We put special focus on the comparison of patients treated with 5-fluorouracil/cisplatin (‘Walsh’) or carboplatin/paclitaxel (‘CROSS’). We compared characteristics between patients treated according to ‘Walsh’ vs. ‘CROSS’. Cox regression was performed to test for an association of parameters with outcomes. Study eligibility was met by 90 patients. First, the higher age and more comorbidities of the ‘CROSS’ patients, along with a shorter intensive care/intermediate care stay, might reflect an improvement in supportive and surgical/perioperative procedures over the periods. Second, the ‘CROSS’ patients experienced more hematologic toxicity and were less likely to complete chemotherapy as per protocol. This indicates that efforts should be taken to guide patients through a toxic treatment regimen by supportive measures. Third, the negative prognostic impact of radiochemotherapy-related toxicities (i.e., dysphagia and hematologic toxicities) and the duration of the intensive care/intermediate care unit stay underlines that further optimization of treatment procedures remains an important goal. We found no differences in tumor downstaging and survival between treatment regimen. Toxicity profiles could be improved by tailoring the regimen to individual patients (e.g., careful use of the taxane-based regimen in elderly patients).

## 1. Introduction

Esophageal cancer is often diagnosed at advanced stages (i.e., in Germany, UICC stages III-IV in about 70%) [1,2]. Recent reports provide poor 5-year overall survival (OS) rates of 20% for these patients [1]. Worldwide, squamous cell cancers represent about 87% of all esophageal cancers [3]. The poor survival and, during the last decades unchanged, constantly high mortality rates, demonstrate the need for more effective treatment approaches [1]. Here, several clinical trials introduced preoperative radiochemotherapy (RCT) followed by surgery for locally advanced esophageal cancer [4,5,6]. Around the turn of the millennium, the RCT regimen including 5-fluorouracil/cisplatin (introduced by Walsh et al. [4]) was widely adopted [7,8,9]. In 2012, van Hagen et al. presented the results of the ‘CROSS’ trial [10]. Since then, the RCT regimen with carboplatin/paclitaxel has been widely adopted [11]. Randomized trials for the comparison of both regimens are lacking. Beyond this, numerous approaches during the past decades have aimed to improve outcomes after multimodality treatment. These included modern radiotherapy techniques, further development of surgical techniques, and recently, the evaluation of targeted therapies and immune checkpoint inhibitors as part of the multimodal concepts [12,13,14,15,16]. 

In the light of the aforementioned progress and developments, we retrospectively studied treatment results, toxicities, surgical outcomes, and prognostic factors in patients with squamous cell esophageal cancer treated with preoperative RCT and surgery at our institution. Here, to take into account the major shift in treatment strategies, we put a special focus on the differences in treatment according to ‘Walsh’ and ‘CROSS’.

## 2. Results

### 2.1. Patient Baseline, Radiochemotherapy, Surgical, and Histopathological Characteristics

A total of 90 patients were eligible for our study (Figure S1, flowchart). These patients were treated during the period of 01/1999 to 02/2019. Among them, 63 patients (70%) were treated according to the ‘Walsh protocol’, and 27 patients (30%) were treated according to the ‘CROSS protocol’. The cohort consisted of 67 male patients (74.4%) and 23 female patients (25.6%). Median age was 63.0 years (range, 42.3–79.5 years). In our clinic, we standardly assessed patients for 54 months. Due to patient’s death or loss to follow-up, in the present study, we have a median follow-up of 28.1 months (range, 2.1–165.9 months). Since the majority of tumor-related events occur within the first two years after diagnosis, this follow-up time seems adequate to draw conclusions [17]. In 75 patients (83.3%), an abdominothoracic esophagus resection was performed. In these patients, additional simultaneous surgical procedures were a partial lung resection (*n* = 3), and a combined partial resection of lung and liver (*n* = 1). Here, during pre-surgical staging examinations (*n* = 2 patients) or intraoperative examination (*n* = 1 patient), organ lesions were evident and were excised for further evaluation. In histopathological examination, these lesions were benign. In one patient, a partial lung resection was necessary due to adhesions after previous surgical interventions. In 15 patients (16.7%), an esophagectomy with cervical anastomosis was performed. An R0 resection was achieved in all of the patients. Please see Table 1 for the distribution of the patient cohort depending on the RCT regimen. The patient groups differed statistically significant in terms of age, Charlson Comorbidity Index, and radiotherapy technique.

### 2.2. Toxicity, Treatment Compliance and Surgical Outcomes

Radiotherapy was completed without treatment interruptions in all patients. In total, 67/90 patients (74.4%) received 100% of the planned chemotherapy cycles and dose, whereas significantly less patients in the carboplatin/paclitaxel group completed chemotherapy (Table 2). The reasons for chemotherapy dose reduction, skip, or cessation in patients treated with 5-fluorouracil/cisplatin were: Leucopenia (*n* = 6), decrease in creatinine clearance (*n* = 3), infection (*n* = 1), deterioration of hearing (*n* = 1), and combined deterioration of hearing and decrease in creatinine clearance (*n* = 1). The reasons in patients treated with carboplatin/paclitaxel were: Leucopenia (*n* = 8), deterioration of clinical condition (*n* = 2), infection (*n* = 1), and elevated liver enzymes (*n* = 1). The rates of hematologic toxicity (≥grade 3) were higher in the carboplatin/paclitaxel group. Furthermore, we analyzed whether patients who experienced RCT-related toxicities were less likely to receive full chemotherapy. Indeed, the rates of patients with incomplete chemotherapy administration were significantly higher (*p* < 0.05, Chi-square test, data not included in Table 2) in the group of patients with hematologic and/or acute organ toxicity ≥grade 3 (16/38 patients, 42.1%) patients) than in patients with hematologic and/or acute organ toxicity <grade 3 (8/52 patients, 15.4%). In total, 45/88 patients (51.1%, data missing for 2 patients) experienced postsurgical complications. The intensive/intermediate care unit stay was significantly longer in the 5-fluorouracil/cisplatin group than in the carboplatin/paclitaxel group. Death within 30 days post-surgery occurred in only 2/90 patients (2.2%). Table 2 and Table 3 and Table S1 summarize details on patient distributions and surgical complications.

### 2.3. Survival Outcomes and Prognostic Factors

**Figure 1:**
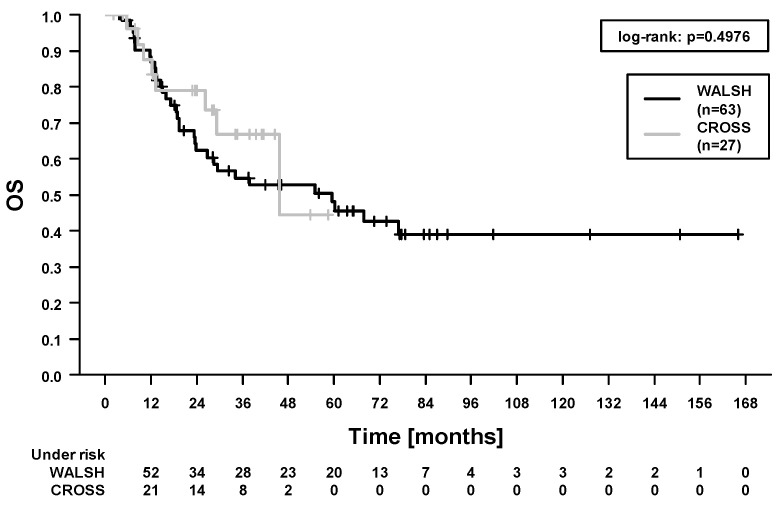
Overall survival (OS). Comparison between patients treated with carboplatin/paclitaxel (‘CROSS’) and patients treated with 5-fluorouracil/cisplatin (‘WALSH’).

**Figure 2:**
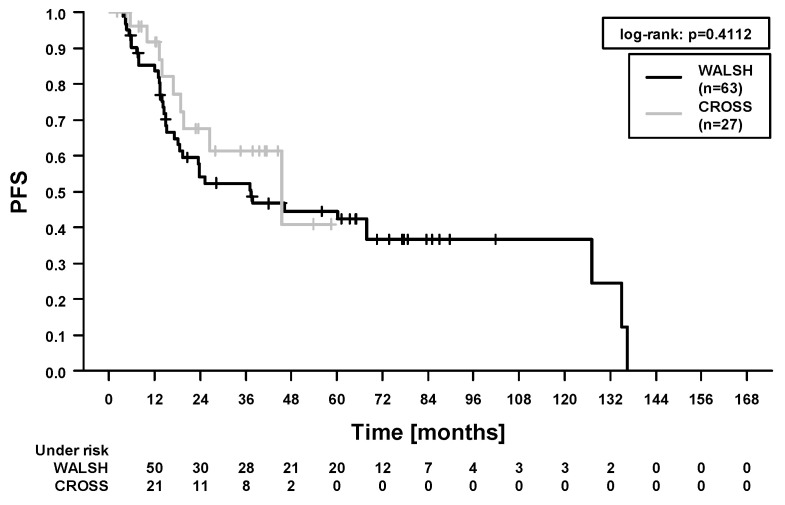
Progression-free survival (PFS). Comparison between patients treated with carboplatin/paclitaxel (‘CROSS’) and patients treated with 5-fluorouracil/cisplatin (‘WALSH’).

**Figure 3:**
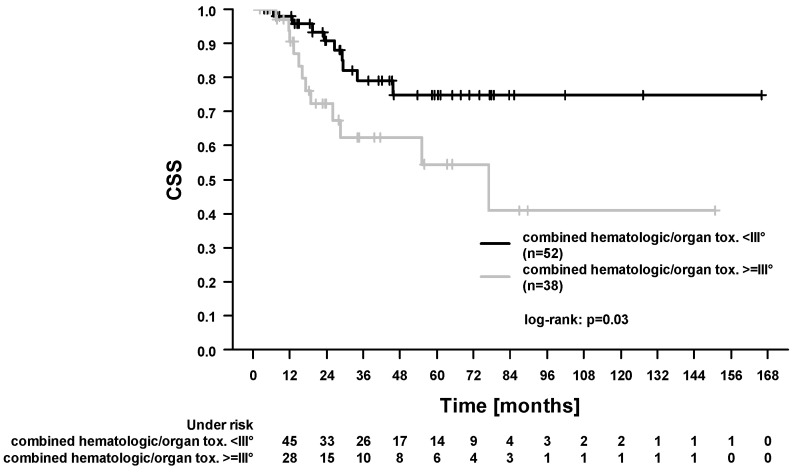
Cancer-specific survival (CSS). Comparison between patients who experienced hematologic and/or acute organ toxicity ≥grade 3 and patients with toxicities <grade 3.

**Figure 4:**
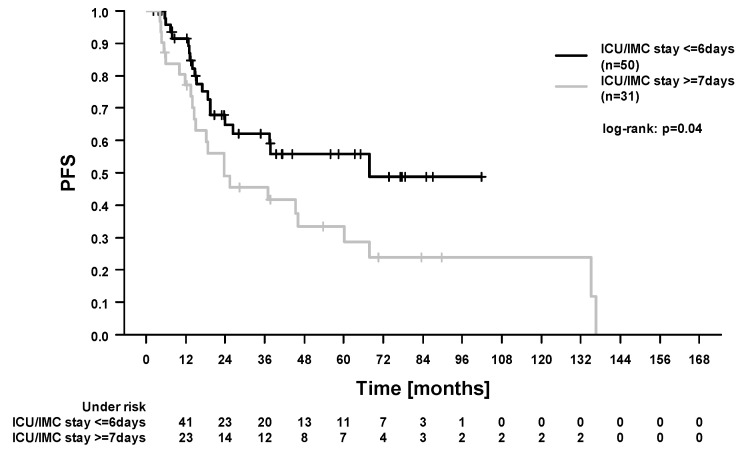
Progression-free survival (PFS). Comparison of survival according to the stay on intensive care/intermediate care unit (ICU/IMC).

In the whole study cohort, the 5-year locoregional control (LRC), progression-free survival (PFS), OS, and cancer-specific survival (CSS) were 84.1%, 46.2%, 49.7%, and 66.9%. In total, 40/90 patients (44.4%) died during follow-up. Among these, 21 patients died from esophageal cancer. In 19 patients, the cause of death was not further specified in the medical records. A total of 10/90 patients (11.1%) developed locoregional recurrence. Here, 4 patients had combined local and regional recurrences, 4 patients had isolated local recurrences, and 2 patients had isolated regional recurrences. In our study, 21/90 patients (23.3%) developed distant metastases. In the Cox regression analysis (Table 4), there was no difference in survival outcomes between patients treated with carboplatin/paclitaxel and patients treated with 5-fluorouracil/cisplatin (Figure 1 and Figure 2, OS and PFS). We found significantly improved outcomes in patients with pathological complete response (for LRC, PFS, OS, and CSS). We found significantly worse outcomes in patients who experienced acute organ toxicity ≥grade 3 (for PFS and CSS), in patients with dysphagia ≥grade 3 (for CSS), in patients with combined acute hematologic and/or organ toxicity ≥grade 3 (for CSS, Figure 3), and in patients with longer stay on intensive care/intermediate care unit (for PFS and CSS, Figure 4). 

## 3. Discussion

The outcome in patients with locally advanced esophageal cancer was significantly improved by the introduction of preoperative RCT [4,10,18]. The gain in survival times was especially beneficial for squamous cell cancers [18]. However, randomized trials have not yet compared the RCT regimen introduced by Walsh et al. and van Hagen et al. [4,10]. Currently, the ‘National Comprehensive Cancer Network’ guidelines give a category 1 recommendation for both regimens [19]. There have only been few retrospective studies, which could not consistently demonstrate a superiority for either regimen [8,20,21,22]. Thus, this issue certainly remains of scientific interest [8]. Beyond this, numerous advances were achieved in local and systemic treatment [12,13,16]. Nevertheless, despite optimal treatment under study conditions, in the ‘CROSS’ trial, 211/366 patients (57.7%) experienced tumor progression (either locoregional and/or distant) during follow-up [18]. This demonstrates the need to further optimize treatment strategies and guidance. Here, the understanding of clinical courses in non-trial patients’ daily routine (including toxicities, complications, and prognostic factors) can be helpful. Thus, we retrospectively analyzed outcomes at our institution with particular focus on the different RCT regimens.

In the present study, we compared different RCT regimens and, thus, have to consider the esophageal cancer incidence and local treatment practice in the periods 1999–2014 (treatment according to the ‘Walsh’ trial) and 2014–2019 (treatment according to the ‘CROSS’ trial). Remarkably, we found patients treated with carboplatin/paclitaxel to be almost 10 years older (median 70.1 vs. 61.1 years) than patients treated with 5-fluorouracil/cisplatin. First, this might reflect the general tendency that the tumor incidence rises at the age of ≥60 years, whereas it declines in younger patients [1]. Second, a shift to elderly patients could reflect the refinement of oncologic treatment modalities (e.g., surgery, perioperative management, radiotherapy, and supportive therapeutics [12,13,23,24]) during the period. Through this, clinicians might have tended to plan multimodal treatment including preoperative RCT and surgery in elderly patients. 

Additionally, we found a shorter intensive care/intermediate care unit stay (median 4 vs. 7 days) in patients treated with carboplatin/paclitaxel. This finding is especially remarkable, since these patients were about 10 years older and had more comorbidities. This might reflect recent developments and efforts to improve surgical techniques and perioperative procedures [25]. Overall, our study’s complication rates (51.1% of the patients) and 30-day mortality (2.2%) were comparable with larger datasets. Low et al. reported a complication rate of 59% in 2704 esophagectomies [26], and van Hagen et al. reported a 30-day mortality of 2–3% (depending on the study arm) in the ‘CROSS’ trial [10]. Both, the shift to older and more comorbid patients with, at the same time, shorter intensive/intermediate care unit stay, and the favorable complication and mortality rates in our study might be attributed to the treatment in a high-volume cancer center [27]. However, when comparing the ‘Walsh’ and ‘CROSS’ regimen, we found no differences in other surgical or perioperative parameters (i.e., surgical technique, surgical complications, hospital stay). In general, previously published studies did not find any consistent differences in these endpoints between the ‘Walsh’ and ‘CROSS’ protocol [8,28]. In summary, this can be understood as a confirmation of the good feasibility of either RCT regimen [18,29].

Furthermore, we found that VMAT was used more frequently in patients treated with carboplatin/paclitaxel (3/27 patients, 11.1%) than in patients treated with 5-fluorouracil/cisplatin (0/63 patients). This, again, represents technical developments and innovations in local therapies, whereas, with a limited number of patients, the results should not be overinterpreted. Here, the question may arise why we did not treat more patients with intensity-modulated radiotherapy (IMRT) or VMAT in the respective period. Based on previous reports of our clinic [30] and other authors [31], there was evidence that even low doses of radiation to the lung (≥5Gy, [31]) could increase the rates of lung complications. As previous authors speculated, IMRT or VMAT might increase the lung volume exposed to this low dose of irradiation [22]. This could be a rationale for the reluctant use of IMRT or VMAT in our study. In the meantime, numerous studies reported a good feasibility of IMRT and/or VMAT for preoperative RCT of esophageal cancer [12,32]. A direct evidence for an increase in pulmonary complications was not shown [12,32]. Hence, IMRT and/or VMAT were used more frequently in other contemporary studies which compared different regimen: Münch et al. treated 71–100% (depending on the regimen) with VMAT, Sanford et al. treated 2.6–29.9% with IMRT (depending on the regimen) [8,28]. Though most patients were treated with 3DCRT in our study, the rates of acute and late organ toxicities were relatively low. In total, 26/90 patients (28.9%) experienced acute toxicity ≥grade 3, and late toxicity ≥grade 3 occurred in none of the patients. A treatment break or a dose reduction of radiotherapy was not necessary for any of the patients. It can be assumed that these low rates of toxicities along with high treatment compliance leave sufficient space for an effective systemic treatment and for surgery.

In our study, we found higher rates of hematologic toxicities for the ‘CROSS’ regimen. In contrast, Münch et al. found the ‘Walsh’ regimen to be associated with higher rates of hematologic toxicities [8]. These findings might possibly be explained by differing median patient age in the study populations. We report a median age of 70.1 years for the patients treated according to the ‘CROSS’ trial, whereas Münch et al. reported a median age of 62 years for the intention-to-treat population [8]. Thus, due to higher age, we might have observed higher rates of hematologic toxicities with carboplatin/paclitaxel. Similarly, Huang et al. demonstrated increased hematologic toxicity with a taxane-based regimen in elderly patients treated with definitive RCT for esophageal cancer [20]. An underlying mechanism might be the decrease in the function of the bone marrow with age [20,33]. Another possible explanation for higher rates of hematologic toxicities in patients treated from 2014–2019 (‘CROSS’) in comparison to patients treated from 1999–2014 (‘Walsh’) might be an improvement in patient care (e.g., with more frequent blood sampling and, thus, a more frequent detection of anemia, leukopenia, and/or thrombopenia). Taken together, the data provide evidence that elderly patients are at an increased risk of hematologic toxicities and that they should be monitored carefully during RCT [34]. 

Additionally, we found that patients who experienced acute organ and/or hematologic toxicities ≥grade 3 were less likely to receive the complete chemotherapy and had worse outcomes (CSS) than patients who experienced toxicities <grade 3. This might be attributed to the reduced compliance to chemotherapy due to hematologic toxicities (as demonstrated for patients with anal cancer [35]). However, we could not demonstrate a direct influence of the completeness of chemotherapy on survival. Here, the retrospective study design and the limited number of patients should be considered. In our study, 21/90 patients (23.3%) developed distant metastases during follow-up. Van Hagen et al. reported that 39% of the patients suffered from distant metastases during follow-up in the ‘CROSS’ trial [18]. It is important to note that the comparison is limited by differences in the studies (i.e., retrospective vs. prospective design, inclusion of squamous cell cancers, and adenocarcinomas in the ‘CROSS’ trial). Taken together, efforts should be taken to intensify systemic treatment and to prevent distant spread [36]. This could be realized by an optimization of supportive therapeutics to ensure the application of the complete chemotherapy [35] and/or by the integration of further agents like immune checkpoint inhibitors which may exert a synergistic effect with RCT [14,37].

Furthermore, we found worse outcomes (for CSS) in patients who experienced dysphagia ≥grade 3 during RCT. Previous authors reported that the tumor length is an independent prognostic factor in esophageal cancer [38]. Furthermore, tumor length was described to be predictive for esophagitis during RCT [39]. Hence, the worse prognosis in patients who experienced dysphagia could reflect the greater tumor length. Additionally, dysphagia could lead to a worse nutritional status and, consequently, negatively affect postoperative outcomes [40,41]. Here, in our study, we found that a longer stay on the intensive care/intermediate care unit was associated with worse survival (PFS and CSS). Similarly, Rasmussen et al. found postoperative complications to have negative prognostic impact [42]. The mentioned associations imply that efforts should be made to optimize local treatment. In detail, modern radiotherapy techniques [12] or the further development of clinical pathways in the perioperative setting [25] could relevantly improve outcomes. 

Finally, we found local treatment with either RCT regimen to be comparably effective. In the presented study, only 10/90 patients (11.1%) developed locoregional recurrence. In the whole study population, we found a pathological complete response in 35/90 patients (38.9%). In line with previous studies, our results underscore the prognostic relevance of pathological complete response [43]. For this item, we found a significant association with each survival endpoint in the Cox regression analysis, with hazard ratios between 0.08 and 0.25. In patients treated according to the ‘CROSS’ trial, we found a relatively low rate of pathological complete responses (33.3%) compared to patients with squamous cell carcinomas in the ‘CROSS’ trial (49%) [10]. This might be attributed to the facts that, in our study, patients presented with cN+ tumors more often (85.2% vs. 65% of the patients in the ‘CROSS’ trial). Additionally, they received the full dose of chemotherapy less frequently (55.6% vs. 91% of the patients in the ‘CROSS’ trial) [10]. Finally, in our study, tumor downstaging (ypT/ypN stages) and survival were comparable for the RCT according to ‘Walsh’ and ‘CROSS’. However, as depicted in the survival curves, there was a relevant number of censored patients. This is an important limitation of the study and conclusions should be drawn cautiously. In summary, either RCT regimen was comparably effective in terms of tumor control. At the same time, the ‘CROSS’ patients experienced more hematologic toxicity and were less likely to complete chemotherapy as per protocol. Hence, our study indicates that the ‘Walsh’ regimen could be advantageous over the ‘CROSS’ regimen in preoperative RCT for esophageal squamous cell cancer. Additionally, our study indicates that toxicity profiles could be improved by tailoring the chemotherapy regimen to individual patients (e. g., careful use of the taxane-based regimen in elderly patients) [20].

## 4. Patients and Methods

### 4.1. Study Design and Patient Eligibility

We studied the medical records of patients with esophageal cancer who were treated at our Department of Radiotherapy and Radiation Oncology. We included patients who received a preoperative RCT (5-fluorouracil/cisplatin or carboplatin/paclitaxel) and subsequent tumor resection for squamous cell esophageal cancer. Patients had adequate organ functions and performance status (ECOG 0-1) to undergo preoperative RCT. They were deemed operable by experienced surgeons at our University Medical Center during interdisciplinary tumor conferences. Additionally, the indication for preoperative RCT was set in the multidisciplinary tumor board. The staging procedures and the therapeutic management were based on the respective contemporary guidelines [29,44,45]. The study was conducted according to the guidelines of the Declaration of Helsinki, and approved by the Ethics Committee of the University of Göttingen Medical Center (protocol code 23/6/20, 17 June 2020).

### 4.2. Multimodal Treatment: Preoperative Radiochemotherapy and Surgical Procedures

Initially, in our clinic, the RCT regimen was based on the trial published by Walsh et al. [4]. From 2014, the regimen of the ‘CROSS’ trial was introduced as a new standard [10]. The RCT procedures were partly described by previous authors [9,30,46]. Patients were treated with 6 MV and/or 20 MV photons. The planning CT scan was acquired with at least 5mm slice thickness. When the tumors were located in the distal esophagus, the renal clearance was assessed separately for each kidney with scintigraphy. The gross tumor volume, defined according to radiological and endoscopic findings, included the primary tumor and the involved lymph nodes. The clinical target volume was generated by manually expanding the volume by 4cm (in case of proximity to larynx or stomach, 2 cm) in craniocaudal directions, including the whole circumference of the esophagus. The planning target volume resulted when a 1cm isotropic margin was added. The system Eclipse (Varian Medical Systems, Palo Alto, CA, USA) was used for treatment planning. Patients standardly received 40 Gy in 2 Gy fractions or 41.4 Gy in 1.8 Gy fractions. If 3D conformal radiotherapy (3DCRT) was applied, an anterior-posterior field arrangement was used. If necessary, additional lateral or oblique fields were added. In our patient cohort, a volumetric modulated arc therapy (VMAT) was rarely used (*n* = 3). For both 3D conformal radiotherapy and VMAT, the lung constraint was V (5 Gy) ≤ 30%. Before initiation of RCT, the isocentre and (for 3D conformal radiotherapy) the fields were simulated with a conventional x-ray simulator using oral contrast agent.

The chemotherapy regimen comprised 2 cycles (in the first and fifth week of RCT) of 5-fluorouracil (15 mg/kg/d over 5 days) and cisplatin (75 mg/m^2^ on day 7) or 5 weekly cycles of carboplatin (area under the curve of 2mg/mL*min on day 1) and paclitaxel (50 mg/m^2^ on day 1). The pretreatment examinations included blood cell counts and clinical chemistry analyses, an electrocardiogram, and the assessment of the 24-h urine creatinine clearance. The prophylaxis of nausea and vomiting was conducted according to the MASCC/ESMO guidelines [47].

Surgery was planned 6 weeks after RCT. Patients underwent thorough re-staging including diagnostic endoscopy. Additionally, patients were assessed by an experienced surgeon and were discussed in the multidisciplinary tumor board again. The surgical procedures were conducted according to the contemporary guidelines [27,29,45]. Pathological assessment of tumor regression was performed according to Mandard et al. [48].

### 4.3. Patient Monitoring during RCT and Follow-up

Patients were hospitalized during the days of the chemotherapy application. During outpatient radiotherapy, weekly clinical and laboratory examinations were conducted. Acute toxicity was scored using the CTCAE criteria v. 5.0 [49], the late toxicity was scored using the LENT/SOMA criteria [50]. The follow-up procedures were performed according to contemporary guidelines [29,44,45]. In the radiotherapy department, patients were standardly assessed for 54 months (every 18 months) during follow-up. Additionally, a more frequent follow-up was regularly performed by the treating gastroenterologist or surgeon.

### 4.4. Statistical Analysis

The comparisons of patient and disease baseline characteristics, toxicities, and complications were performed by Chi-square test and Kruskal–Wallis test. The survival times were considered beginning at the date of histopathological tumor diagnosis. The endpoints for the LRC were both local and regional recurrences. The PFS was considered as the time to tumor progression (both locoregional and distant) or death from any cause. The endpoint for CSS was patient death caused by esophageal cancer progression. The survival curves were created using Kaplan–Meier statistics. The comparisons of survival times for the different groups were performed using log-rank statistics and Cox regression analysis. The software Statistica (v. 13), SPSS (v. 26), and R (v. 4.0.2) with the plugin ‘KWWin’ [51] were used. We considered *p*-values < 0.05 as statistically significant.

## 5. Conclusions

First, we compared the characteristics of patients treated with carboplatin/paclitaxel and with 5-fluorouracil/cisplatin. The patients treated according to the ‘CROSS’ trial had a shorter intensive care/intermediate care unit stay. This might reflect an improvement in supportive and surgical/perioperative procedures in the different time periods. Second, patients treated with carboplatin/paclitaxel experienced more hematologic toxicities and were less likely to receive the complete chemotherapy. This indicates that efforts should be taken to carefully guide patients through a toxic treatment regimen by supportive measures [35]. Additionally, the integration of immune checkpoint inhibitors could be approaches to ensure the prevention of distant metastases [14]. Third, in the whole cohort, we found RCT-related toxicities (primarily, dysphagia, and hematological toxicities) and the need for intensive care/intermediate care to be negative prognostic factors. Thus, further optimization of RCT and surgical/perioperative procedures remains an important goal. In summary, either RCT regimen (‘Walsh’ and ‘CROSS’) was comparably effective in terms of tumor control. At the same time, the ‘CROSS’ patients experienced more hematologic toxicity and were less likely to complete chemotherapy as per protocol. Hence, our study indicates that the ‘Walsh’ regimen could be advantageous over the ‘CROSS’ regimen. Our study indicates that toxicity profiles could be improved by tailoring the chemotherapy regimen to individual patients (e.g., careful use of the taxane-based regimen in elderly patients) [20].

## Figures and Tables

**Table 1:** Baseline patient and disease characteristics and histopathological characteristics. For each parameter, if not otherwise specified, the number of patients and, in brackets, the percentage are given. The statistical comparisons were performed with Chi-square test and Kruskal–Wallis test. ECOG: Eastern Cooperative Oncology Group. 3DCRT: 3D conformal radiotherapy. VMAT: Volumetric modulated arc therapy.

Characteristics	5‑Fluorouracil/ Cisplatin (*n* = 63)	Carboplatin/ Paclitaxel (*n* = 27)	*p*-Value
Age, years, median (min, max)	61.1 (42.3–77.7)	70.1 (43.7–79.5)	<0.01
ECOG performance status			0.96
0	47 (74.6)	20 (74.1)	
1	16 (25.4)	7 (25.9)	
Charlson Comorbidity Index			0.02
<4	23 (36.5)	3 (11.1)	
≥4	40 (63.5)	24 (88.9)	
Female	13 (20.6)	10 (37.0)	0.10
Follow-up, months, median (min, max)	28.6 (3.6–165.9)	26.2 (2.1–58.4)	0.12
Behavioral factors			0.09
Smoking w/o regular alcohol	22 (34.9)	14 (51.9)	
Alcohol abuse w/o smoking	6 (9.5)	1 (3.7)	
Smoking and alcohol abuse	18 (28.6)	6 (22.2)	
Neither smoking nor regular alcohol	15 (23.8)	5 (18.5)	
Undetermined	2 (3.2)	1 (3.7)	
Tumor localization			0.28
Upper third	2 (3.2)	1 (3.7)	
Middle third	29 (46.0)	12 (44.4)	
Lower third	32 (50.8)	14 (51.9)	
T category clinical/ultrasound			0.77
T1	1 (1.6)	1 (3.7)	
T2	6 (9.5)	8 (29.6)	
T3	51 (81.0)	17 (63.0)	
T4	5 (7.9)	1 (3.7)	
Nodal status clinical/ultrasound			0.76
N0	11 (17.5)	4 (14.8)	
N+	52 (82.5)	23 (85.2)	
UICC stage [AJCC 8th edition, 2017]			0.1
II	14 (22.2)	12 (44.4)	
III	44 (70.0)	14 (51.9)	
IVA	5 (7.8)	1 (3.7)	
Radiotherapy dose, median (min, max)	40.0 (39.6–41.4)	41.4 (41.4–41.4)	
Radiotherapy technique			<0.01
3DCRT	63 (100.0)	24 (88.9)	
VMAT	0 (0.0)	3 (11.1)	
Surgical technique			0.73
Abdominothoracic esophagus resection	52 (82.5)	23 (85.2)	
Esophagectomy with cervical anastomosis	11 (17.5)	4 (14.8)	
ypT stage			0.28
ypT0	28 (44.4)	12 (44.4)	
ypT1	2 (3.2)	4 (14.9)	
ypT2	11 (17.5)	5 (18.5)	
ypT3	21 (33.3)	6 (22.2)	
ypT4	1 (1.6)	0 (0.0)	
ypN stage			0.09
ypN0	48 (76.2)	16 (59.3)	
ypN1	13 (20.6)	7 (25.9)	
ypN2	2 (3.2)	4 (14.8)	
Resection status: R0	63 (100)	27 (100)	
Pathological complete response	26 (41.3)	9 (33.3)	0.48

**Table 2:** Radiochemotherapy: Treatment completion rates and acute toxicity. For each parameter, the number of patients and, in brackets, the percentage are given. The statistical comparisons were performed with Chi-square test and Kruskal–Wallis test.

Characteristics	5‑Fluorouracil/ Cisplatin (*n* = 63)	Carboplatin/ Paclitaxel (*n* = 27)	*p*-Value
Radiotherapy			-
Received the planned dose without treatment breaks	63 (100)	27 (100)	
Chemotherapy			0.01
Received 100% of the planned cycles and dose	51 (81.0)	15 (55.6)	
Received < 100% of the planned cycles and/or dose	12 (19.0)	12 (44.4)	
Acute toxicity			
Overall acute organ toxicity			0.26
≥grade 3	16 (25.4)	10 (37.0)	
<grade 3	47 (74.6)	17 (63.0)	
Mucositis			0.74
0	61 (96.8)	24 (88.9)	
1	1 (1.6)	1 (3.7)	
2	0	1 (3.7)	
3	1 (1.6)	1 (3.7)	
Dermatitis			0.58
0	49 (77.8)	25 (92.6)	
1	13 (20.6)	2 (7.4)	
2	1 (1.6)	0	
Nausea			0.75
0	46 (73.0)	21 (77.8)	
1	11 (17.5)	2 (7.4)	
2	6 (9.5)	3 (11.1)	
3	0	1 (3.7)	
Dysphagia			0.18
0	2 (3.2)	1 (3.7)	
1	13 (20.6)	8 (29.6)	
2	30 (47.6)	7 (25.9)	
3	15 (23.8)	11 (40.7)	
4	3 (4.8)	0	
Hematologic toxicity			
Overall hematologic toxicity			0.04
≥grade 3	11 (17.5)	10 (37.0)	
<grade 3	52 (82.5)	17 (63.0)	
Anaemia			0.40
0	10 (15.9)	4 (14.8)	
1	43 (68.3)	18 (66.7)	
2	6 (9.5)	4 (14.8)	
3	4 (6.3)	1 (3.7)	
Leukopenia			0.29
0	18 (28.6)	3 (11.1)	
1	21 (33.3)	7 (25.9)	
2	17 (27.0)	7 (25.9)	
3	6 (9.5)	9 (33.3)	
4	1 (1.6)	1 (3.7)	
Thrombopenia			0.53
0	40 (63.5)	17 (63.0)	
1	20 (31.7)	8 (29.6)	
2	2 (3.2)	1 (3.7)	
3	1 (1.6)	1 (3.7)	

**Table 3:** Surgical outcomes and late toxicity. For each parameter, if not otherwise specified, the number of patients and, in brackets, the percentage are given. The statistical comparisons were performed using Chi-square test and Kruskal–Wallis test. Please note that the data for surgical complications are missing in 2 patients. Please see Supplemental Table S1 for a detailed list of surgical complications. The data for the hospital and intensive/intermediate care unit stay are missing for 9 patients. The data for late toxicity are missing for 6 patients.

Characteristics	5‑Fluorouracil/ Cisplatin (*n* = 63)	Carboplatin/ Paclitaxel (*n* = 27)	*p*-Value
Surgical outcomes			
Surgical complications			0.43
Yes	30 (48.4)	15 (57.7)	
No	32 (51.6)	11 (42.3)	
Hospital stay, days, median (min, max)	19 (7–87)	15 (8–140)	0.24
Intensive/intermediate care unit stay, days, median (min, max)	7 (1–54)	4 (1–115)	0.01
Death within 30 days post-surgery	1 (1.6)	1 (3.7)	0.53
Late toxicity			
Late dysphagia			0.58
0	55 (87.3)	18 (85.7)	
1	6 (9.5)	3 (11.1)	
2	2 (3.2)	0	
Dermatitis			0.52
0	62 (98.4)	27 (100.0)	
1	1 (1.6)	0	

**Table 4:** Univariate Cox regression analysis including patient baseline, tumor- and treatment- related variables, and toxicities and complications. The hazard ratios and 95% confidence intervals are given. *p* values < 0.05 were considered as statistically significant. LRC: Locoregional control, PFS: Progression-free survival, OS: Overall survival, CSS: Cancer-specific survival, pCR: Pathological complete response, RCT: Radiochemotherapy, ICU/IMC: Intensive care unit/intermediate care unit.

Variable	LRC		PFS		OS		CSS	
	Hazard Ratio (95% CI)	*p* Value	Hazard Ratio (95% CI)	*p* Value	Hazard Ratio (95% CI)	*p* Value	Hazard Ratio (95% CI)	*p* Value
Age (per year)	1.04 (0.98–1.12)	0.23	1.00 (0.97–1.03)	0.88	1.01 (0.98–1.04)	0.55	1.0 (0.96–1.05)	0.85
T stage: u/cT3-4 (74) vs. u/cT1-2 (16)	0.88 (1.87–4.16)	0.87	1.36 (0.57–3.22)	0.49	1.89 (0.67–5.31)	0.23	0.93 (0.31–2.76)	0.89
cN stage: cN+ (75) vs. cN0 (15)	1.69 (0.21–13.39)	0.62	0.92 (0.41–2.08)	0.85	0.87 (0.38–1.97)	0.74	0.46 (0.18–1.19)	0.11
Chemotherapy								
Carboplatin/paclitaxel (27) vs. 5-fluorouracil/cisplatin (63)	2.19 (0.59–8.18)	0.24	0.73 (0.35–1.54)	0.41	0.76 (0.35–1.68)	0.50	0.50 (0.01–1.71)	0.27
Chemotherapy complete: yes (66) vs. no (24)	1.46 (0.38–5.69)	0.58	1.49 (0.78–2.86)	0.23	1.49 (0.76–2.95)	0.25	1.73 (0.69–4.31)	0.24
pCR: yes (35) vs. no (55)	0.08 (0.01–0.66)	0.02	0.25 (0.13–0.50)	<0.01	0.19 (0.08–0.42)	<0.01	0.14 (0.04–0.48)	<0.01
Acute organ toxicity ≥ III°: yes (26) vs. no (64)	2.43 (0.68–8.71)	0.17	1.94 (1.06–3.56)	0.03	1.78 (0.93–3.42)	0.08	2.58 (1.08–6.15)	0.03
Maximal dysphagia ≥ III° during RCT: yes (29) vs. no (61)	2.75 (0.79–9.64)	0.11	1.67 (0.91–3.04)	0.09	1.36 (0.71–2.63)	0.35	3.47 (1.45–8.29)	<0.01
Acute hematologic/organ toxicity ≥ III°: yes (38) vs. no (52)	1.30 (0.37–4.64)	0.69	1.54 (0.86–2.76)	0.15	1.52 (0.82–2.85)	0.19	2.58 (1.06–6.13)	0.03
ICU/IMC stay: ≥7 (31) vs. ≤6 (50) days	1.34 (0.36–4.99)	0.67	1.87 (1.01–3.49)	<0.05	1.78 (0.93–3.39)	0.08	2.56 (1.05–6.28)	0.04

## Data Availability

The datasets generated and/or analyzed in the current study are available from the corresponding author by reasonable request.

## Supplementary Materials

The following are available online at https://www.mdpi.com/article/10.3390/cancers13081834/s1, Table S1: Details for surgical complications. Figure S1: Flowchart. Selection of patients treated with preoperative radiochemotherapy and surgery for esophageal squamous cell cancer.

## References

[1] Kaatsch P., Spix C., Katalinic A., Hentschel S., Luttmann S., Waldeyer-Sauerland M., Waldmann A., Christ M., Folkerts J., Hansmann J. (2020). Cancer in Germany in 2015/2016.

[2] Zhang Y. (2013). Epidemiology of esophageal cancer. World J. Gastroenterol..

[3] Uhlenhopp D.J., Then E.O., Sunkara T., Gaduputi V. (2020). Epidemiology of esophageal cancer: Update in global trends, etiology and risk factors. Clin. J. Gastroenterol..

[4] Walsh T.N., Noonan N., Hollywood D., Kelly A., Keeling N., Hennessy T.P. (1996). A comparison of multimodal therapy and surgery for esophageal adenocarcinoma. N. Engl. J. Med..

[5] Burmeister B.H., Smithers B.M., Gebski V., Fitzgerald L., Simes R.J., Devitt P., Ackland S., Gotley D.C., Joseph D., Millar J. (2005). Surgery alone versus chemoradiotherapy followed by surgery for resectable cancer of the oesophagus: A randomised controlled phase III trial. Lancet Oncol..

[6] Stahl M. (2010). Is there any role for surgery in the multidisciplinary treatment of esophageal cancer?. Ann. Oncol..

[7] Ilson D.H. (2008). Esophageal cancer chemotherapy: Recent advances. Gastrointest Cancer Res..

[8] Münch S., Pigorsch S.U., Feith M., Slotta-Huspenina J., Weichert W., Friess H., Combs S.E., Habermehl D. (2017). Comparison of neoadjuvant chemoradiation with carboplatin/ paclitaxel or cisplatin/ 5-fluoruracil in patients with squamous cell carcinoma of the esophagus. Radiat. Oncol..

[9] Hennies S., Hermann R.M., Gaedcke J., Grade M., Hess C.F., Christiansen H., Wolff H.A. (2014). Increasing toxicity during neoadjuvant radiochemotherapy as positive prognostic factor for patients with esophageal carcinoma. Dis. Esophagus.

[10] van Hagen P., Hulshof M.C., van Lanschot J.J., Steyerberg E.W., van Berge Henegouwen M.I., Wijnhoven B.P., Richel D.J., Nieuwenhuijzen G.A., Hospers G.A., Bonenkamp J.J. (2012). Preoperative chemoradiotherapy for esophageal or junctional cancer. N. Engl. J. Med..

[11] Paireder M., Jomrich G., Kristo I., Asari R., Rieder E., Beer A., Ilhan-Mutlu A., Preusser M., Schmid R., Schoppmann S.F. (2020). Modification of preoperative radiochemotherapy for esophageal cancer (CROSS protocol) is safe and efficient with no impact on surgical morbidity. Strahlenther Onkol..

[12] Münch S., Aichmeier S., Hapfelmeier A., Duma M.N., Oechsner M., Feith M., Combs S.E., Habermehl D. (2016). Comparison of dosimetric parameters and toxicity in esophageal cancer patients undergoing 3D conformal radiotherapy or VMAT. Strahlenther Onkol..

[13] Mariette C., Markar S.R., Dabakuyo-Yonli T.S., Meunier B., Pezet D., Collet D., D’Journo X.B., Brigand C., Perniceni T., Carrere N. (2019). Hybrid Minimally Invasive Esophagectomy for Esophageal Cancer. N. Engl. J. Med..

[14] Liao X.Y., Liu C.Y., He J.F., Wang L.S., Zhang T. (2019). Combination of checkpoint inhibitors with radiotherapy in esophageal squamous cell carcinoma treatment: A novel strategy. Oncol. Lett..

[15] Abdo J., Agrawal D.K., Mittal S.K. (2017). “Targeted” Chemotherapy for Esophageal Cancer. Front. Oncol..

[16] Li C., Zhao S., Zheng Y., Han Y., Chen X., Cheng Z., Wu Y., Feng X., Qi W., Chen K. (2021). Preoperative pembrolizumab combined with chemoradiotherapy for oesophageal squamous cell carcinoma (PALACE-1). Eur. J. Cancer.

[17] Lindenmann J., Fediuk M., Fink-Neuboeck N., Porubsky C., Pichler M., Brcic L., Anegg U., Balic M., Dandachi N., Maier A. (2020). Hazard Curves for Tumor Recurrence and Tumor-Related Death Following Esophagectomy for Esophageal Cancer. Cancers.

[18] Shapiro J., van Lanschot J.J.B., Hulshof M., van Hagen P., van Berge Henegouwen M.I., Wijnhoven B.P.L., van Laarhoven H.W.M., Nieuwenhuijzen G.A.P., Hospers G.A.P., Bonenkamp J.J. (2015). Neoadjuvant chemoradiotherapy plus surgery versus surgery alone for oesophageal or junctional cancer (CROSS): Long-term results of a randomised controlled trial. Lancet Oncol..

[19] NCCN Esophageal and Esophagogastric Junction Cancers. https://www.nccn.org/professionals/physician_gls/PDF/esophageal.pdf.

[20] Huang C., Huang D., Zhu Y., Xie G., Wang H., Shi J., Jia B., Yuan Y., Zhang W. (2020). Comparison of a Concurrent Fluorouracil-Based Regimen and a Taxane-Based Regimen Combined with Radiotherapy in Elderly Patients with Esophageal Squamous Cell Carcinoma. Transl. Oncol..

[21] Jiang D.M., Sim H.W., Espin-Garcia O., Chan B.A., Natori A., Lim C.H., Moignard S., Chen E.X., Liu G., Darling G. (2021). Chemoradiotherapy Using Carboplatin plus Paclitaxel versus Cisplatin plus Fluorouracil for Esophageal or Gastroesophageal Junction Cancer. Oncology..

[22] Haisley K.R., Hart K.D., Nabavizadeh N., Bensch K.G., Vaccaro G.M., Thomas C.R., Schipper P.H., Hunter J.G., Dolan J.P. (2017). Neoadjuvant chemoradiotherapy with concurrent cisplatin/5-fluorouracil is associated with increased pathologic complete response and improved survival compared to carboplatin/paclitaxel in patients with locally advanced esophageal cancer. Dis. Esophagus.

[23] Graham L., Wikman A. (2016). Toward improved survivorship: Supportive care needs of esophageal cancer patients, a literature review. Dis. Esophagus.

[24] Watanabe M., Okamura A., Toihata T., Yamashita K., Yuda M., Hayami M., Fukudome I., Imamura Y., Mine S. (2018). Recent progress in perioperative management of patients undergoing esophagectomy for esophageal cancer. Esophagus.

[25] Preston S.R., Markar S.R., Baker C.R., Soon Y., Singh S., Low D.E. (2013). Impact of a multidisciplinary standardized clinical pathway on perioperative outcomes in patients with oesophageal cancer. Br. J. Surg..

[26] Low D.E., Kuppusamy M.K., Alderson D., Cecconello I., Chang A.C., Darling G., Davies A., D’Journo X.B., Gisbertz S.S., Griffin S.M. (2019). Benchmarking Complications Associated with Esophagectomy. Ann. Surg..

[27] Hoeppner J., Plum P.S., Buhr H., Gockel I., Lorenz D., Ghadimi M., Bruns C., Qualitätskommission der Deutschen Gesellschaft für Allgemein- und Viszeralchirurgie (2021). Surgical treatment of esophageal cancer-Indicators for quality in diagnostics and treatment. Chirurg.

[28] Sanford N.N., Catalano P.J., Enzinger P.C., King B.L., Bueno R., Martin N.E., Hong T.S., Wo J.Y., Mamon H.J. (2017). A retrospective comparison of neoadjuvant chemoradiotherapy regimens for locally advanced esophageal cancer. Dis. Esophagus.

[29] Lordick F., Mariette C., Haustermans K., Obermannova R., Arnold D., Committee E.G. (2016). Oesophageal cancer: ESMO Clinical Practice Guidelines for diagnosis, treatment and follow-up. Ann. Oncol..

[30] Dähn D., Martell J., Vorwerk H., Hess C.F., Becker H., Jung K., Hilgers R., Wolff H.A., Hermann R.M., Christiansen H. (2010). Influence of irradiated lung volumes on perioperative morbidity and mortality in patients after neoadjuvant radiochemotherapy for esophageal cancer. Int. J. Radiat. Oncol. Biol. Phys..

[31] Wang S.L., Liao Z., Vaporciyan A.A., Tucker S.L., Liu H., Wei X., Swisher S., Ajani J.A., Cox J.D., Komaki R. (2006). Investigation of clinical and dosimetric factors associated with postoperative pulmonary complications in esophageal cancer patients treated with concurrent chemoradiotherapy followed by surgery. Int. J. Radiat. Oncol. Biol. Phys..

[32] Martini S., Arcadipane F., Strignano P., Spadi R., Contu V., Fiandra C., Ragona R., Catalano G., Satolli M.A., Camandona M. (2018). Volumetric modulated arc therapy (VMAT) in the treatment of esophageal cancer patients. Med. Oncol..

[33] Minami H., Ohe Y., Niho S., Goto K., Ohmatsu H., Kubota K., Kakinuma R., Nishiwaki Y., Nokihara H., Sekine I. (2004). Comparison of pharmacokinetics and pharmacodynamics of docetaxel and Cisplatin in elderly and non-elderly patients: Why is toxicity increased in elderly patients?. J. Clin. Oncol..

[34] Wakui R., Yamashita H., Okuma K., Kobayashi S., Shiraishi K., Terahara A., Sasano N., Ohtomo K., Nakagawa K. (2010). Esophageal cancer: Definitive chemoradiotherapy for elderly patients. Dis. Esophagus.

[35] Glynne-Jones R., Meadows H.M., Lopes A., Muirhead R., Sebag-Montefiore D., Adams R., ACTII Study Group (2020). Impact of compliance to chemoradiation on long-term outcomes in squamous cell carcinoma of the anus: Results of a post hoc analysis from the randomised phase III ACT II trial. Ann. Oncol..

[36] Wu S.G., Zhang W.W., Sun J.Y., Li F.Y., Lin Q., He Z.Y. (2018). Patterns of Distant Metastasis Between Histological Types in Esophageal Cancer. Front. Oncol..

[37] Yang H., Wang K., Wang T., Li M., Li B., Li S., Yuan L. (2020). The Combination Options and Predictive Biomarkers of PD-1/PD-L1 Inhibitors in Esophageal Cancer. Front. Oncol..

[38] Wu J., Chen Q.X. (2016). Prognostic and predictive significance of tumor length in patients with esophageal squamous cell carcinoma undergoing radical resection. BMC Cancer.

[39] Werner-Wasik M., Yorke E., Deasy J., Nam J., Marks L.B. (2010). Radiation dose-volume effects in the esophagus. Int. J. Radiat. Oncol. Biol. Phys..

[40] van der Schaaf M.K., Tilanus H.W., van Lanschot J.J., Johar A.M., Lagergren P., Lagergren J., Wijnhoven B.P. (2014). The influence of preoperative weight loss on the postoperative course after esophageal cancer resection. J. Thorac. Cardiovasc. Surg..

[41] Yoshida N., Baba Y., Shigaki H., Harada K., Iwatsuki M., Kurashige J., Sakamoto Y., Miyamoto Y., Ishimoto T., Kosumi K. (2016). Preoperative Nutritional Assessment by Controlling Nutritional Status (CONUT) is Useful to estimate Postoperative Morbidity After Esophagectomy for Esophageal Cancer. World J. Surg..

[42] Rasmussen S.R., Nielsen R.V., Fenger A.S., Siemsen M., Ravn H.B. (2018). Postoperative complications and survival after surgical resection of esophageal squamous cell carcinoma. J. Thorac. Dis..

[43] Meredith K.L., Weber J.M., Turaga K.K., Siegel E.M., McLoughlin J., Hoffe S., Marcovalerio M., Shah N., Kelley S., Karl R. (2010). Pathologic response after neoadjuvant therapy is the major determinant of survival in patients with esophageal cancer. Ann. Surg. Oncol..

[44] Malthaner R.A., Wong R.K., Rumble R.B., Zuraw L., Gastrointestinal Cancer Disease Site Group of Cancer Care Ontario’s Program in Evidence-based, C (2004). Neoadjuvant or adjuvant therapy for resectable esophageal cancer: A clinical practice guideline. BMC Cancer.

[45] Porschen R., Fischbach W., Gockel I., Hollerbach S., Holscher A., Jansen P.L., Miehlke S., Pech O., Stahl M., Thuss-Patience P. (2019). S3-Leitlinie—Diagnostik und Therapie der Plattenepithelkarzinome und Adenokarzinome des Ösophagus. Z. Gastroenterol..

[46] Hermann R.M., Horstmann O., Haller F., Perske C., Christiansen H., Hille A., Schmidberger H., Fuzesi L. (2006). Histomorphological tumor regression grading of esophageal carcinoma after neoadjuvant radiochemotherapy: Which score to use?. Dis. Esophagus.

[47] Roila F., Herrstedt J., Aapro M., Gralla R.J., Einhorn L.H., Ballatori E., Bria E., Clark-Snow R.A., Espersen B.T., Feyer P. (2010). Guideline update for MASCC and ESMO in the prevention of chemotherapy- and radiotherapy-induced nausea and vomiting: Results of the Perugia consensus conference. Ann. Oncol..

[48] Mandard A.M., Dalibard F., Mandard J.C., Marnay J., Henry-Amar M., Petiot J.F., Roussel A., Jacob J.H., Segol P., Samama G. (1994). Pathologic assessment of tumor regression after preoperative chemoradiotherapy of esophageal carcinoma. Clinicopathologic correlations. Cancer.

[49] U.S. Department of Health and Human Services, N.I.o.H., National Cancer Institute Common Terminology Criteria for Adverse Evens (CTCAE), Version 5.0. https://ctep.cancer.gov/protocoldevelopment/electronic_applications/docs/CTCAE_v5_Quick_Reference_8.5x11.pdf.

[50] Rubin P., Constine L.S., Fajardo L.F., Phillips T.L., Wasserman T.H. (1995). RTOG Late Effects Working Group. Overview. Late Effects of Normal Tissues (LENT) scoring system. Int. J. Radiat. Oncol. Biol. Phys..

[51] Gross A., Ziepert M., Scholz M. (2012). KMWin—A convenient tool for graphical presentation of results from Kaplan-Meier survival time analysis. PLoS ONE.

