# Supplementary material for: Preoperative Radiochemotherapy in Esophageal Squamous Cell Cancer with 5-Fluorouracil/Cisplatin or Carboplatin/Paclitaxel: Treatment Practice over a 20-Year Period and Implications for the Individual Treatment Modalities
Source: Cancers (Basel). 2021 Apr 12;13(8):1834. doi: 10.3390/cancers13081834 (PMC8068912; doi:10.3390/cancers13081834)
Supplement: Supplementary file 1 [file cancers-13-01834-s001.zip › Suppl. Figure S1_revision 3.pptx]

## Slide 1
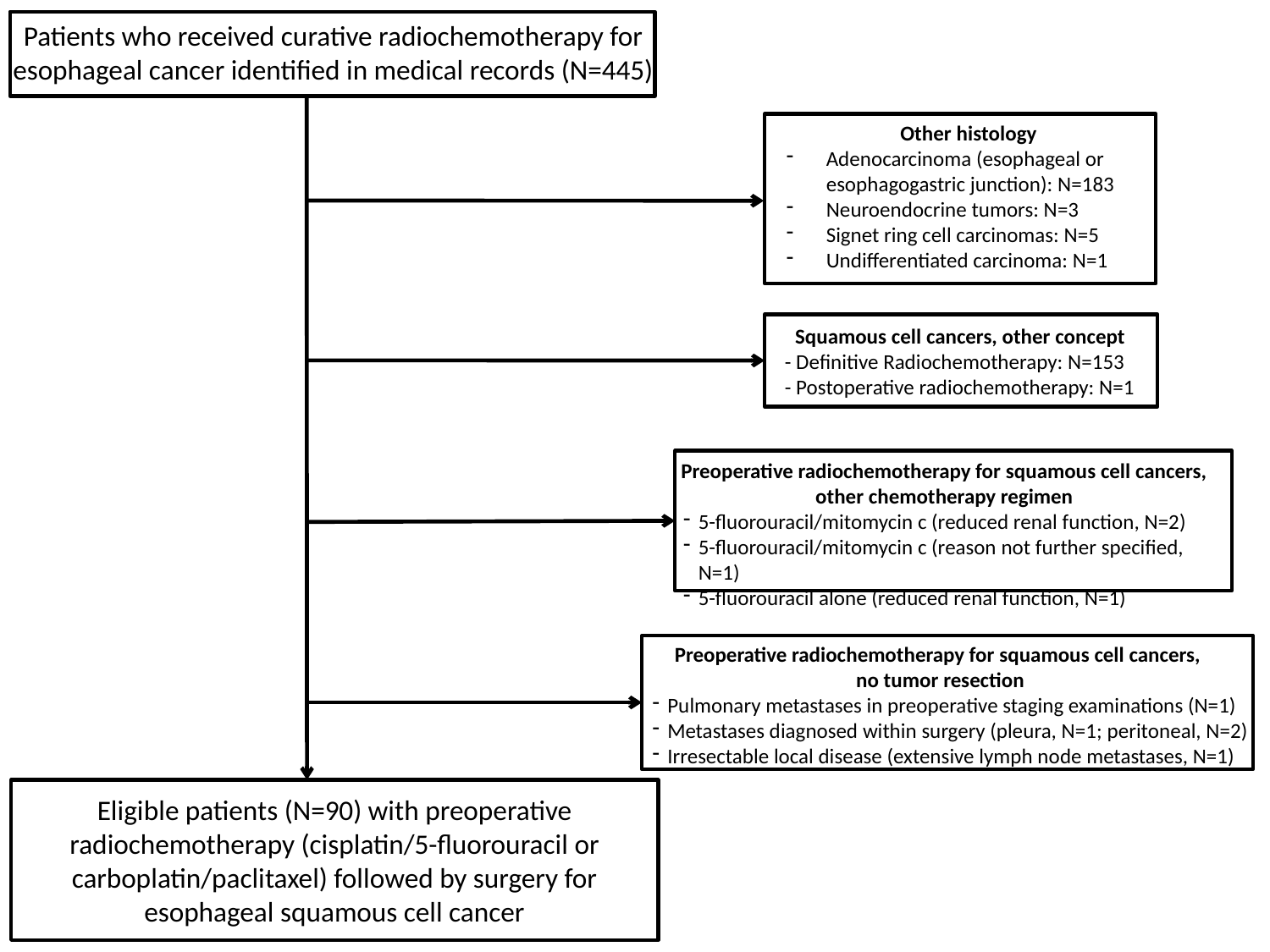

Patients who received curative radiochemotherapy for esophageal cancer identified in medical records (N=445)
Squamous cell cancers, other concept
- Definitive Radiochemotherapy: N=153
- Postoperative radiochemotherapy: N=1
Preoperative radiochemotherapy for squamous cell cancers, other chemotherapy regimen
5-fluorouracil/mitomycin c (reduced renal function, N=2)
5-fluorouracil/mitomycin c (reason not further specified, N=1)
5-fluorouracil alone (reduced renal function, N=1)
Eligible patients (N=90) with preoperative radiochemotherapy (cisplatin/5-fluorouracil or carboplatin/paclitaxel) followed by surgery for esophageal squamous cell cancer
Other histology
Adenocarcinoma (esophageal or esophagogastric junction): N=183
Neuroendocrine tumors: N=3
Signet ring cell carcinomas: N=5
Undifferentiated carcinoma: N=1
Preoperative radiochemotherapy for squamous cell cancers,
no tumor resection
Pulmonary metastases in preoperative staging examinations (N=1)
Metastases diagnosed within surgery (pleura, N=1; peritoneal, N=2)
Irresectable local disease (extensive lymph node metastases, N=1)
