# Supplementary material for: Preoperative Radiochemotherapy in Esophageal Squamous Cell Cancer with 5-Fluorouracil/Cisplatin or Carboplatin/Paclitaxel: Treatment Practice over a 20-Year Period and Implications for the Individual Treatment Modalities
Source: Cancers (Basel). 2021 Apr 12;13(8):1834. doi: 10.3390/cancers13081834 (PMC8068912; doi:10.3390/cancers13081834)
Supplement: Supplementary file 1 [file cancers-13-01834-s001.zip › Suppl. Table S1.docx]

| **Suppl. Table S1. Details for surgical complications.** | |
| --- | --- |
| **Type of complication** | **Number of patients** |
| Anastomotic leakage | 16 |
| Anastomotic stenosis | 2 |
| Bleeding | 3 |
| Cardiac arrhythmia | 4 |
| Central venous catheter infection | 1 |
| Chyle fistula | 7 |
| Death | 1 |
| Cardiac complications | 2 |
| Perforation of the gastric tube | 1 |
| Pleural empyema | 1 |
| Pneumonia | 11 |
| Thoracic wall emphysema | 2 |
| Tracheoesophageal fistula | 1 |
| Vocal cord palsy | 6 |
| Wound healing disorder | 6 |
